# A Newborn with Simmering Bleeding after Circumcision

**DOI:** 10.7759/cureus.3324

**Published:** 2018-09-18

**Authors:** Lars Mense, Emanuela Ferretti, Raveena Ramphal, Thierry Daboval

**Affiliations:** 1 Neonatology, University Hospital Carl Gustav Carus, Dresden, DEU; 2 Pediatrics, Children's Hospital of Eastern Ontario, Ottawa, CAN; 3 Haematology / Oncology, Children's Hospital of Eastern Ontario, Ottawa, CAN; 4 Paediatrics, Children's Hospital of Eastern Ontario, Ottawa, CAN

**Keywords:** hemophilia, circumcision, bleeding disorder, newborn, coagulation factors

## Abstract

We present a case of a healthy male neonate born at term, circumcised on Day 1 of life. Facing ongoing bleeding at the incision site, the baby was transferred to a level III neonatal intensive care unit for further investigation and management. His family history was unremarkable for bleeding disorders. On arrival, the baby was hemodynamically stable with abnormal coagulation values. Further investigations revealed a diagnosis compatible with severe hemophilia A. He deteriorated on Day 2, developing acute severe anemia which required two red blood cell transfusions. This rare but potentially fatal event reminds clinicians to remain extremely vigilant with minor surgical procedures such as circumcision even in the absence of family history.

## Introduction

Diagnosing inherited bleeding disorders in newborns can be difficult including interpretation of preliminary blood results and identifying the cause of the bleeding [1]. Whether bleeding in a healthy newborn, who has had a minor procedure, is considered abnormal is often a judgement call and can cause anxiety to parents and health care providers. Clinicians need to remain extremely vigilant for bleeding disorders after minor surgical procedures. We present a case of a healthy male neonate born at term who was circumcised on his first day of life and developed persistent bleeding at the surgical site. The patient had a negative family history for coagulopathy but was later diagnosed with hemophilia A. The differential diagnosis of abnormal bleeding in a newborn is reviewed and treatment is discussed.

## Case presentation

After an unremarkable pregnancy and negative family history for bleeding, a male term neonate (39+5 weeks of gestation, birth weight 3295 grams) was born via spontaneous vaginal delivery at a level II hospital. He did not require resuscitation. He received one dose of vitamin K intramuscularly (IM) as per recommendation [2] without bleeding complications. The mother mentioned later that she had noted persisting bleeding after heel pricks.

**Figure 1:**
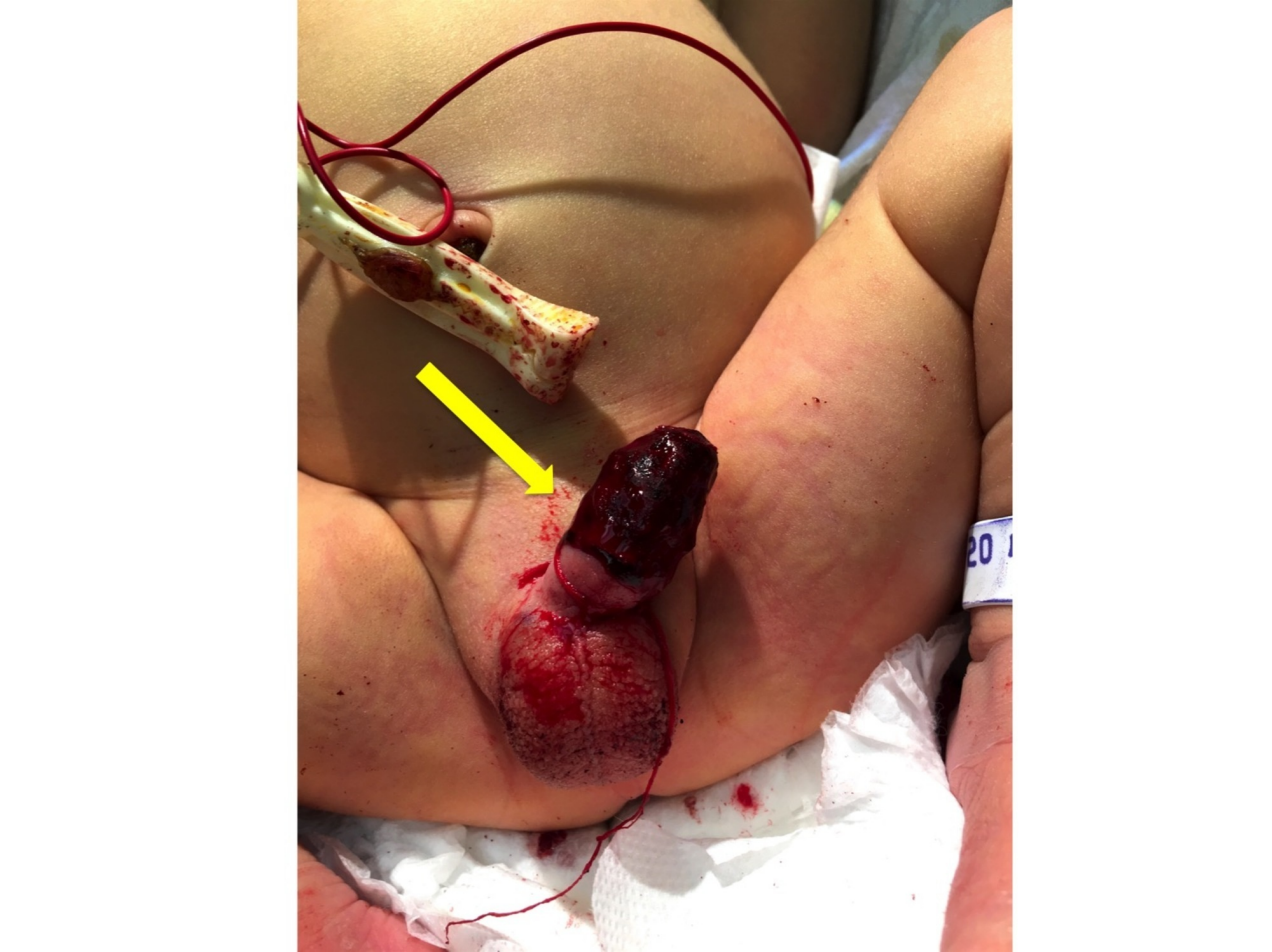
Surgical site on patient admission. Simmering bleeding is noted. Yellow arrow shows the surgical site.

Parents opted to circumcise their son for social reasons. Persistent bleeding complicated the procedure done on Day 1 of life. Repeated sutures to stop the bleeding were ineffective, however the baby stayed hemodynamically stable. The following day, the baby was transferred to a level III neonatal intensive care unit for further management. On admission, the physical examination was unremarkable except for some fresh blood noted at the surgical site (Figure 1) from a diffusely simmering bleed and hematomas in the typical locations for blood sampling. The amount of blood loss was difficult to assess with certainty given the high absorption capacity of the diapers. We therefore remained vigilant for significant bleeding by regularly checking the hemoglobin level. In keeping with earlier observations, the baby developed significant hematomas after blood work and intravenous (IV) access; however, there was no evidence of ecchymosis or muscle hematoma at the site of the vitamin K intra-muscular injection. Blood work showed: hemoglobin 142 g/L (reference range: 135–200 g/L), platelets 139 x 109/L (reference range: 150–600 x 109/L), prothrombin time (PTT) > 150s (reference range: 25.0–60.0s), prothrombin time (PT) international normalized ratio 1.07 (reference range: 0.80–1.16).

Initially, the estimated blood loss was not concerning and further surgical interventions did not seem helpful given the poorly circumscribed origin of the bleeding. The bleeding seemed to slow down remarkably with sterile dressings and topical tranexamic acid.

Surprisingly, acute severe anemia (hemoglobin 58 g/L) was noted the following morning, but fortunately the baby was clinically and hemodynamically stable. Results of further investigations to explore the prolonged PTT were now available and revealed: Factor VIII (FVIII) < 0.01 IU/ml (reference range: 0.50–1.57 IU/ml), normal values for factor IX, XIII, von Willebrand-antigen and von Willebrand-activity. This was compatible with the diagnosis of severe hemophilia A (FVIII < 0.01 UI/ml).

The newborn received one packed red blood cell (PRBC) transfusion (15 ml/kg) and 250 units (70 IU/kg) of recombinant (genetically produced) FVIII concentrate. The dose was based on the entire vial being administered to prevent wastage of an expensive product. The following day, severe anemia (hemoglobin 72 g/L) was noted again and a second PRBC transfusion was given. Head and abdominal ultrasound ruled out internal hemorrhage. After educating the parents, the baby was discharged home and since then is regularly followed by the hemophilia clinic at the local institution.

Gene investigations revealed that the baby has a nonsense mutation which is associated with a 30% to 40% of developing inhibitors. This is the typical rate of inhibitor development in severe haemophiliac patients [3]. His mother was also found to carry the same mutation. The child is currently two years and seven months old and receives weekly prophylaxis with plasma derived FVIII concentrate. He has no evidence of circulating inhibitors after 87 exposure days to FVIII concentrate.

## Discussion

Hemophilia is a mostly inherited genetic disorder typically inherited through an X chromosome. Hemophilia A (FVIII deficiency) affects <1 in 10,000 people (14.2 per 10,000 males), approximately 2500 Canadians. Hemophilia B (Factor IX (FIX) deficiency) affects 1 in 50,000 people (1 per 20,000 males), approximately 600 Canadians. About 30% of cases are a spontaneous mutation with no family history [3]. The severity of hemophilia A is defined by the amount of FVIII measured (mild 0.05–0.40 IU/ml; moderate 0.01–0.05 IU/ml and severe < 0.01 IU/ml). Muscle and joint bleeding is typical. Administration of the decreased clotting factor allows effective treatment nowadays but can be complicated by the development of antibodies against the infused factor (“inhibitor”) in 25–50% of people with severe FVIII deficiency, 1–2% of people with mild or moderate FVIII deficiency and 1.5–5% of people with severe FIX deficiency [4,5]. Earlier publications suggested an inverse correlation between the age (<6 months) of first exposure and inhibitor formation. However, more recent studies that controlled for known confounders were unable to demonstrate an association with age [6]. Patients at higher risk of developing inhibitors are people with haemophilia with relatives who have inhibitors, those who have large genetic mutations and, people with haemophilia of African and Latino descent [5,7,8]. Historically, infections with HIV and hepatitis C posed a risk of factor treatment but today recombinant concentrates have overcome this hazard.

This case reminds clinicians to be extremely vigilant even with minor surgical procedures to avoid unexpected, potentially fatal, outcomes. Two important observations are worth mentioning:

First, major bleeding after circumcision is a rare event [9] and should always call for a diagnostic workup. The differential diagnoses of abnormal bleeding in a newborn include diseases of cellular and plasmatic coagulation (Table 1). Considering that approximately 30% of hemophilia cases are new mutations, absence of family history for coagulopathy may mislead clinicians and potentially delay the diagnosis. Blood work is not generally necessary before circumcision, but given the short history in a newborn baby, physicians should consider investigations like complete blood cell count, PT, PTT and consulting a pediatric hematologist in the presence of subtle signs of coagulopathy prior to performing even minor surgery. A critical risk-benefit appraisal is essential for decision making in these patients and special precautions to minimize bleeding can be taken if a coagulopathy is diagnosed before surgery and the decision [3] to pursue is made.

**Table 1:** Selected differential diagnoses of abnormal bleeding after neonatal circumcision in a well newborn.

Cause	Diagnoses
Surgical	Local bleeding
Cellular	Neonatal alloimmune thrombocytopenia
	Disseminated intravascular coagulation
	Other forms of thrombocytopenia
	Platelet dysfunction
Plasmatic	Hemophilia A
	Hemophilia B
	Von Willebrand syndrome
	Vitamin K deficiency

Second, the extraordinary absorption capacity of the modern diapers could be really challenging when trying to estimate blood loss volume in such a circumstance. This phenomenon needs to be recognized and could explain the team’s underestimation of blood loss.

Here below some important points for clinicians to remember:

Clinical pearls

·         Minimum workup for coagulopathies should be considered before minor surgical procedures such as neonatal circumcision if subtle clinical signs of bleeding disorders are present even with a negative family history. Mainly male newborns are at higher risk of severe hemophilia.

·         A pediatric hematologist should be consulted to help guide treatment decisions and ensure adequate followup when hemophilia is suspected.

·         Simmering bleeding in superabsorbent diaper might lead to underestimation of blood loss.

## Conclusions

We described a very rare case of neonatal haemophilia A with negative family history for bleeding disorder that presented after what seemed to be a limited but persistent bleed after circumcision. Clinicians need to be extremely attentive in such circumstances to be able to diagnose a bleeding disorder early to prevent more severe complications associated with unrecognized but significant bleeding. Benefits and risks of administration of FVIII should be always discussed with a pediatric hematologist.
